# Rapid dynamic changes of FL.2 variant: A case report of COVID-19 breakthrough infection

**DOI:** 10.1016/j.ijid.2023.11.011

**Published:** 2024-01

**Authors:** Wonderful T. Choga, Gobuiwang Khilly Kurusa (Gasenna), James Emmanuel San, Tidimalo Ookame, Irene Gobe, Mohammed Chand, Badisa Phafane, Kedumetse Seru, Patience Matshosi, Boitumelo Zuze, Nokuthula Ndlovu, Teko Matsuru, Dorcas Maruapula, Ontlametse T. Bareng, Kutlo Macheke, Lesego Kuate-Lere, Labapotswe Tlale, Onalethata Lesetedi, Modiri Tau, Mpaphi B. Mbulawa, Pamela Smith-Lawrence, Mogomotsi Matshaba, Roger Shapiro, Joseph Makhema, Darren P. Martin, Tulio de Oliveira, Richard J. Lessells, Shahin Lockman, Simani Gaseitsiwe, Sikhulile Moyo

**Affiliations:** 1 Botswana Harvard AIDS Institute Partnership, Gaborone, Botswana; 2 School of Allied Health Sciences, Faculty of Health Sciences, Gaborone, Botswana; 3 Centre for Epidemic Response and Innovation (CERI), School of Data Science and Computational Thinking, Stellenbosch University, Stellenbosch, South Africa; 4 Lenmed-Bokamoso Private Hospital, Gaborone, Botswana; 5 KwaZulu-Natal Research Innovation and Sequencing Platform (KRISP), School of Laboratory. Medicine and Medical Sciences, University of KwaZulu-Natal, Durban, South Africa; 6 Ministry of Health and Wellness, Gaborone, Botswana; 7 Diagnofirm Medical Laboratories, Plot 12583, Nyerere Drive MiddleStar, Gaborone, Botswana; 8 National Health laboratory, Gaborone, Botswana; 9 Botswana-Baylor Children's Clinical Centre of Excellence, Gaborone, Botswana; 10 Department of Pediatrics, Baylor College of Medicine, Houston, USA; 11 Department of Immunology and Infectious Diseases, Harvard T.H. Chan School of Public Health, Boston, USA; 12 Institute of Infectious Diseases and Molecular Medicine, Division of Computational Biology, Department of Integrative Biomedical Sciences, University of Cape Town, South Africa; 13 Department of Global Health, University of Washington, Seattle, USA; 14 School of Health Systems and Public Health, University of Pretoria, South Africa; 15 Division of Medical Virology, Faculty of Medicine and Health Sciences, Stellenbosch University, Cape Town, South Africa

**Keywords:** SARS-CoV-2, Evolution, FL.2, Immunocompromised, Botswana

## Abstract

We investigated intra-host genetic evolution using two SARS-CoV-2 isolates from a fully vaccinated (primary schedule x2 doses of AstraZeneca plus a booster of Pfizer), >70-year-old woman with a history of lymphoma and hypertension who presented a SARS-CoV-2 infection for 3 weeks prior to death due to COVID-19. Two full genome sequences were determined from samples taken 13 days apart with both belonging to Pango lineage FL.2: the first detection of this Omicron sub-variant in Botswana. FL.2 is a sub-lineage of XBB.1.9.1. The repertoire of mutations and minority variants in the Spike protein differed between the two time points. Notably, we also observed deletions within the ORF1a and Membrane proteins; both regions are associated with high T-cell epitope density. The internal milieu of immune-suppressed individuals may accelerate SARS-CoV-2 evolution; hence, close monitoring is warranted.

## Highlights

• We observed rapid within-host evolution in the fatal case of Omicron FL.2.
• Low-frequency SARS-CoV-2 mutations observed indicate within-host viral evolution.
• Mutations in the Spike protein differed between two time points thirteen days apart.

## Introduction

Immune responses within the context of SARS-CoV-2 vary. Typically, the virus is cleared within 2 weeks of infection, but immunocompromised individuals can have prolonged infections [1,2]. Weak immune systems increase infection risk and post-COVID-19 clinical sequelae, even post-vaccination [3]. Prolonged infections may facilitate viral evolution, including the evolution of immune-evasive viral variants. SARS-CoV-2 variants of concern (VOCs) such as Alpha, Beta, and Gamma may have originated within the context of long-term infections in immunocompromised individuals [4], [5], [6]. Given the enormous potential global impacts of any future VOCs, it is imperative that genomic surveillance activities prioritize the detection and tracking of any unusual highly mutated SARS-CoV-2 lineages, especially when these are discovered infecting immunocompromised individuals. Here we present a case report of a fatal FL.2 infection of undetermined duration (25 days from diagnosis to death) within an older woman who is immunocompromised with hypertension and a history of lymphoma treatment.

### Case presentation

On April 05, 2023, a 75-year-old female fully vaccinated (AstraZeneca primary series x2 doses plus Pfizer booster dose) presented with fever(≥38°C), and dyspnea and was admitted in the hospital after being confirmed to have severe COVID-19 pneumonia using a rapid antigen test. She had hypoxia with an oxygen saturation on room air of 81%. She had chronic hypertension and a prior history of Follicular Lymphoma which was first diagnosed in 2013 and then received chemotherapy and achieved remission. A relapse of lymphoma and lymphopenia persistence was reported in 2018. She was in remission at the time of presentation. She also reported episodes of cough for a period of 3 months with no COVID-19 tests done during the period. She was exposed to different antibiotics prior to her positive COVID-19 test reported here.

In addition, a computed tomography scan of the chest revealed patchy central and peripheral alveolar densities with an apicobasal gradient. The white-blood-cell count of 15.94 × 10^3^/µl, 94% neutrophils, 2.7% lymphocytes, C-reactive protein 191 mg/l, d-dimer 0.65 µg/ml and lactate dehydrogenase 691 µl were reported.

On the 8^th^ day following SARS-CoV-2 diagnosis (day-08), she was retested, and an ongoing SARS-CoV-2 infection was confirmed by a quantitative real-time reverse-transcriptase–polymerase-chain-reaction(qRT-PCR) assay, a fluorescence PCR method developed by DaAn Gene Co., Ltd. Using a nasopharyngeal swab specimen, the real-time cycle threshold values(qCt-values) for the envelope (E) and nucleocapsid (N) genes were 23.9 and 20.5, respectively. Due to respiratory distress, the patient was commenced on high-flow oxygen and was treated with corticosteroids, an anticoagulant and antibiotics. By day-08, her health had rapidly decompensated, necessitating an urgent upgrade to the intensive care unit level of care. A follow-up computed tomography scan of the chest revealed a persisting severe bilateral pneumonia complicated with pneumomediastinum and subcutaneous emphysema.

On day 17, the patient was initiated on empirical vancomycin and colomycin therapy following clinical deterioration and possible nosocomial sepsis; blood culture results subsequently confirmed the presence of an Acinetobacter spp. She was also diagnosed with oropharyngeal candidiasis and was initiated on x7 days fluconazole.

On day 21 (day-21), a qRT-PCR SARS-CoV-2 test demonstrated a decline in qCt-values for the N (21.6) and E (16.7) genes, indicative of ongoing virus replication. Throughout her admission, the patient did not receive any antiviral agents nor immune-based therapies. Regrettably, the patient died on the 25^th^ day after the initial diagnosis.

The day-08 and day-21 samples were submitted to SARS-CoV-2 sequencing and yielded near-complete genomes with median coverage of 93.95% (93.83-94.07%) and an average depth of coverage of 749.73 (24.96-1474.49) for the day-08 sequences; and 96.4% (92.18-92.56%) with coverage of 7931.40 (6570.68-9292.12) for the day-21 sequences, respectively (Supplementary Figure. 1a and 1b). The midnight protocol for Oxford Nanopore sequencing was utilized as described previously [7]. GridION (Oxford Nanopore Technologies, Oxford, UK) was used for sequencing, and MinKNOW release version was set for demultiplexing and base calling in high accuracy mode. Raw FASTQ files produced for each sample were concatenated and analyzed using Genome Detective (GD) [8]. BAM and consensus FASTA files were obtained from the output of GD. The overall consensus sequences for each sample were created by merging the three FASTA files, and the respective BAM files were used to curate minority variants.

**Figure 1:**
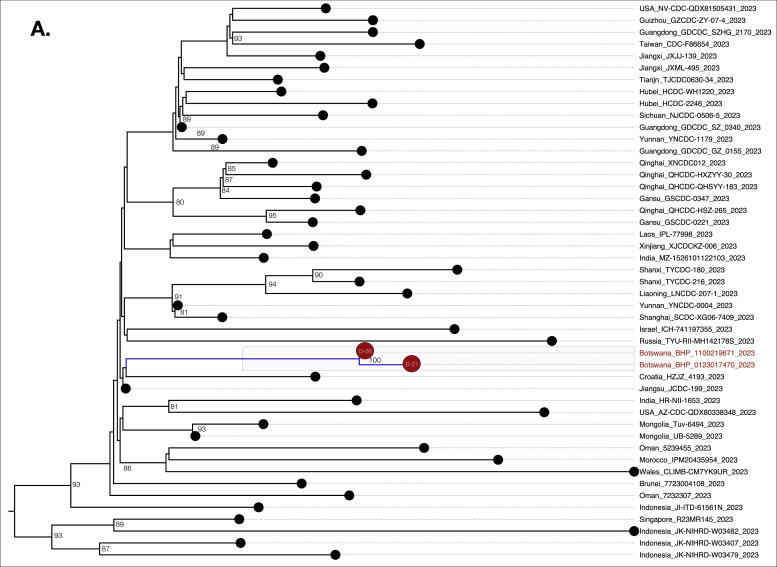

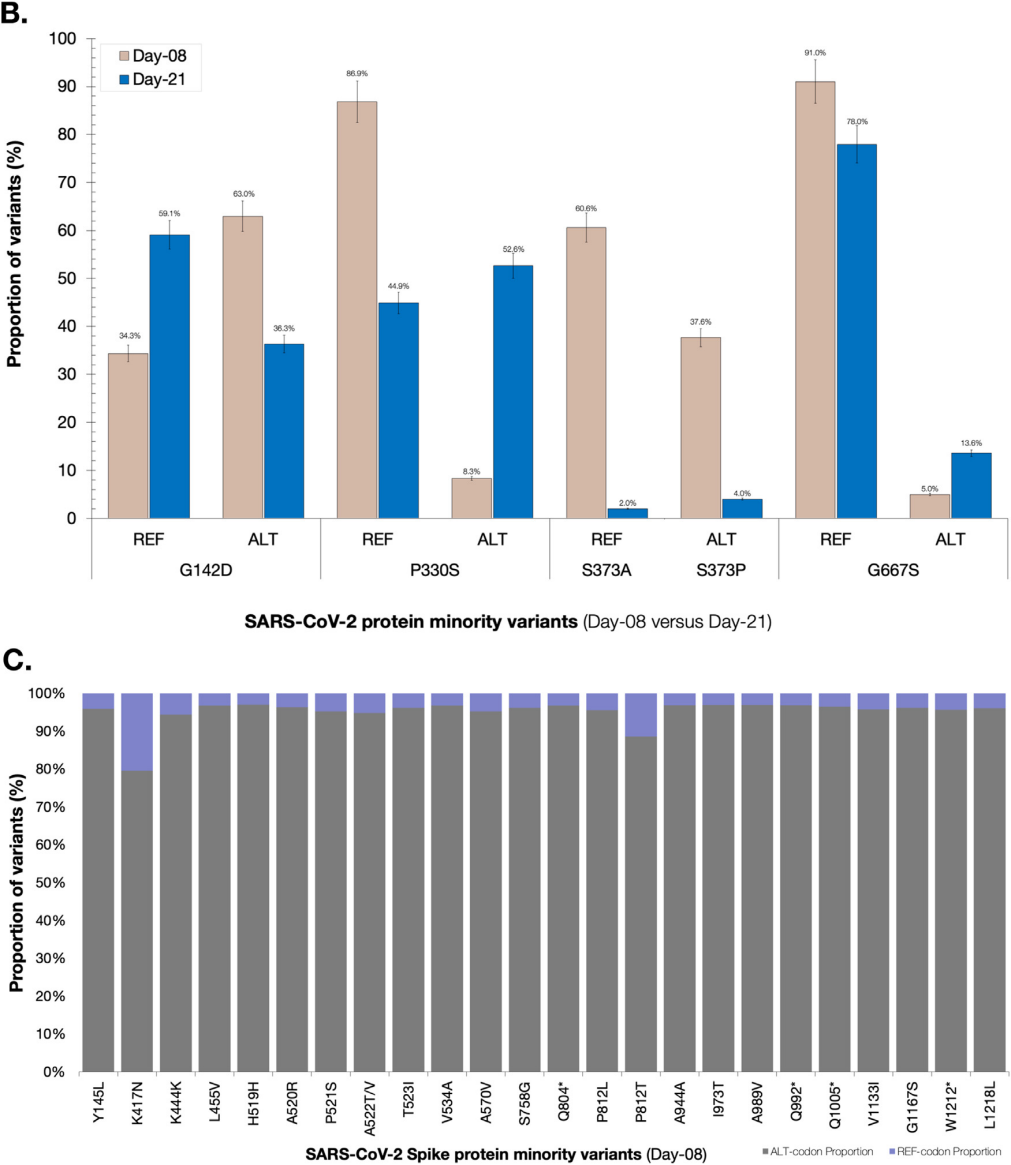

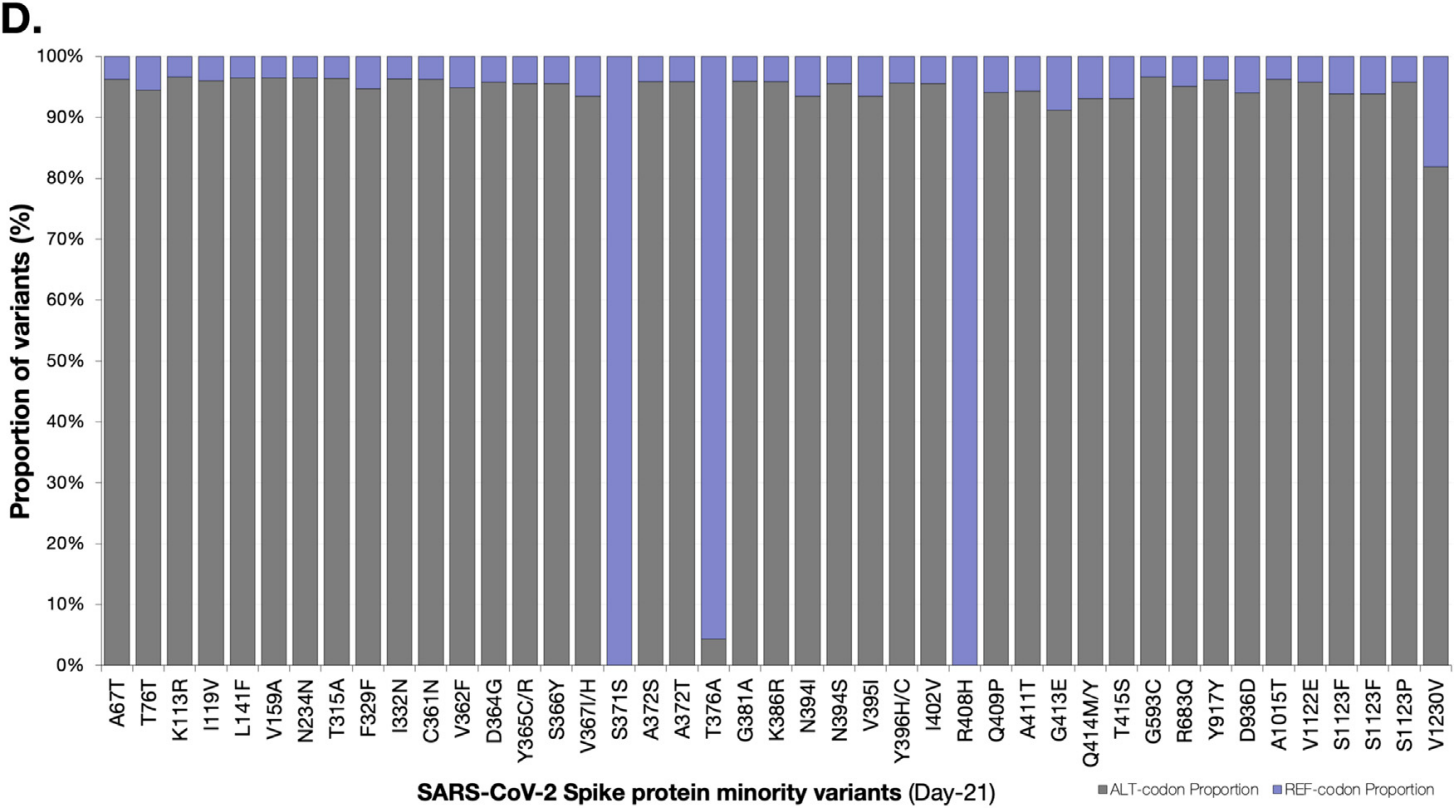
(a) Phylogenetic analysis of three SARS-CoV-2 whole genome sequences from a case with the FL.2 variant under monitoring of SARS-CoV-2. SARS-CoV-2 mutation profiles of Patient 214. The sequences of Patient-214 are represented by color red in the tree. The branches supported with posterior probabilities >0.80 are shown. (b) Distribution of minority variants based on the spike protein and SeqPanther: day-08 vs day-21 variants. (c) Distribution of minority variants based on the spike protein and SeqPanther: day-08 sequence. (d) Distribution of minority variants based on the spike protein and SeqPanther day-21 sequence. * represents a stop-codon.

We genotyped both sequences as FL.2 (https://outbreak.info/); a sublineage of XBB.19.1 variant under monitoring with T4579A [9]. These sequences clustered closest to one another as well as with other global sequences within a larger monophyletic FL.2 clade (Figure 1). This is the first reported case of FL.2 in Botswana. In-depth mutation profiling for the day-08 and day-21 sequences was based on both changes relative to the Wu-H1 (NC_044512) sequence and the FL.2 canonical sequence. Overall, day-08, and day-21 consensus sequences had 96, and 97 substitutions relative to NC_044512 (Supplementary Figure 2).

The day-21 sequence exhibited a rare protein-coding change, P330S [C22550T], present in less than 0.1% (8/6,691) of the FL.2 reported sequences as of July 31, 2023. P330S was at low frequency among variants at day-08 and was not represented at the consensus level (Figure 1b). This mutation occurs within the Spike (S) receptor-binding domain (RBD) (S protein: residues 319-541 residues in the S1 subunit). We also observed reversion at Spike protein position 417 in the day-21 consensus sequence (Figure 1b). The Spike protein of the consensus day-08 sequence had a mutation, K417N (AAT), expected for FL.2 lineage (and all XBB lineages), but the day-21 consensus sequence had an apparent reversion to K (AAG) at this site.

We used SeqPanther [10] with the sequencing depth ≥1000, and minor allele frequency cutoff ≥1% to interrogate the intra-sample viral variant diversity between day-08 and day-21 sequences at genome positions known or strongly suspected to impact the transmissibility and immune evasiveness of SARS-CoV-2. For instance, the minority variant distributions in sequencing reads for the two time points at Spike positions P330, K417, G142, S373, and G667 significantly differed between the time points based on comparisons of proportions (p<0.05; Wilcoxon-Ranksum Test), Figure 1b**;** Supplementary Table 1. Notably, there were minority mutations that were observed exclusively in the Spike protein of either day-08 or day-21 sequences, Figure 1c and 1d, respectively. Based on the SARS-CoV-2 Spike amino-acid finesses S RBD antibody escape calculators (https://jbloomlab.github.io/SARS2-RBD-escape-calc/), the computed ACE2 affinity score of 1.01, and RBD immune escape score of 0.91 indicated that the mutations carried by FL.2 interferes with the neutralization of many NTD-binding monoclonal antibodies without substantially impacting ACE2 binding. Other significant changes were observed in the nsp2, PL^pro^ (nsp3), and Membrane (M) proteins (Supplementary File-1).

## Discussion

We herein report rapid mutation progression of viruses in the FL.2 lineage of the SAR-CoV-2 Omicron VOC in a fully vaccinated older patient who died 21 days following a positive COVID-19 test. The patient remained positive throughout indicating ongoing viral replication. The existence of viral diversity within the patient and their history of immunosuppression are both consistent, but do not definitively prove that the patient had experienced a prolonged infection. Our instance strengthens the argument that rapid and putatively prolonged SARS-CoV-2 infection may lead to immune escape, resulting in the emergence of new variants [1,2]. Prolonged infections have been implicated in the evolution of most SARS-COV-2 VOCs [4], [5], [6],11]. The US Centers for Disease Control observed a 10% increase in the rate of hospitalization since Dec of 2022 when FL.2 was circulating, however at the time of this report, FL.2 was being replaced with Omicron variants EG.5.1 in the USA and BA.2.6 in the rest of the world (https://covid.cdc.gov/covid-data-tracker). Older patients with underlying chronic comorbidities constitute high-risk populations prone to persistent SARS-CoV-2 infections, higher rates of intensive care unit admission, invasive mechanical ventilation, and death [3,^12^,13].

Since there were no available samples prior to April 05, 2023, we cautiously postulate that the present scenario could be attributed to an undiagnosed persistent COVID-19 infection, considering the history of dry cough persisting over a span of 3 months prior to hospitalization of this patient. However, we also do not rule out the possibility of lymphoma involvement in the lungs and that other opportunistic infections may have been present.

The existence of substantial viral diversity within the patient after the 21 days following their admission may be attributable to a variety of factors such as her suppressed immune landscape (despite her vaccination status), or the high replicative capacity of the infecting viruses. An important limitation of our study is that we were not able to evaluate vaccine-induced immune responses in the patient. This, in turn led to our inability to confirm immune dysfunction as credible explanation of the patient's failure to clear the infection. Additionally, the patient was not initiated on any SAR-CoV-2 antiviral therapies after testing positive. This highlights, the importance of using SARS-CoV-2 antiviral drugs among patients who are immunosuppressed, regardless of their SARS-CoV-2 vaccination status, to maximize the chances of complete viral suppression. Most of the minority variants we detected by sequencing at both of the sampling timepoints, including those at Spike positions G142D, P330S, and K417N, have been previously observed in Delta and Omicron variants [14,15]. The mutations occurring in Spike have known impacts on neutralization by NTD-binding monoclonal and polyclonal antibodies, one escape from convalescent sera, and on escape from vaccine-induced immunity [14,15]. Nonetheless, we acknowledge that other variants and deletions detected at low frequencies during sequencing may be attributable to artefacts of the sequencing process and hence should be interpreted with caution. However**,** our findings suggest that the internal environment of immunocompromised patients could be the primary context for the emergence of VOC. Therefore, the findings further emphasize the importance of early diagnosis of SAR-CoV-2 and the use of aggressive antiviral treatments in patients experiencing difficulties in clearing their infections.

## CRediT authorship contribution statement

**Wonderful T. Choga:** Conceptualization, Methodology, Formal analysis, Investigation, Visualization, Data curation, Funding acquisition, Validation, Writing – original draft, Writing – review & editing. **Gobuiwang Khilly Kurusa (Gasenna):** Conceptualization, Investigation. **James Emmanuel San:** Methodology, Validation, Formal analysis, Investigation. **Tidimalo Ookame:** Investigation, Writing – review & editing. **Irene Gobe:** Investigation, Writing – review & editing, Supervision. **Mohammed Chand:** Investigation, Writing – review & editing. **Badisa Phafane:** Investigation, Writing – review & editing. **Kedumetse Seru:** Investigation, Writing – review & editing. **Patience Matshosi:** Investigation, Writing – review & editing. **Boitumelo Zuze:** Investigation, Writing – review & editing. **Nokuthula Ndlovu:** Investigation, Writing – review & editing. **Teko Matsuru:** Investigation, Writing – review & editing. **Dorcas Maruapula:** Writing – review & editing. **Ontlametse T. Bareng:** Investigation, Writing – review & editing. **Kutlo Macheke:** Investigation, Writing – review & editing. **Lesego Kuate-Lere:** Investigation, Writing – review & editing. **Labapotswe Tlale:** Investigation, Writing – review & editing. **Onalethata Lesetedi:** Investigation, Writing – review & editing. **Modiri Tau:** Writing – original draft. **Mpaphi B. Mbulawa:** Writing – review & editing. **Pamela Smith-Lawrence:** Investigation, Writing – review & editing. **Mogomotsi Matshaba:** Investigation. **Roger Shapiro:** Resources, Investigation. **Joseph Makhema:** Investigation, Writing – original draft, Writing – review & editing, Writing – review & editing, Supervision. **Darren P. Martin:** Investigation. **Tulio de Oliveira:** Validation, Investigation, Writing – review & editing, Supervision. **Richard J. Lessells:** Validation, Investigation, Writing – review & editing, Funding acquisition. **Shahin Lockman:** Resources, Investigation, Writing – review & editing, Funding acquisition. **Simani Gaseitsiwe:** Conceptualization, Resources, Investigation, Supervision, Funding acquisition, Project administration, Validation, Writing – review & editing. **Sikhulile Moyo:** Conceptualization, Methodology, Resources, Formal analysis, Investigation, Writing – original draft, Writing – review & editing, Supervision, Funding acquisition.

## Declarations of Competing Interest

The authors have no competing interests to declare.

## Appendix Supplementary materials

Image, application 1

Image, application 2

Image, application 3
