# Supplementary material for: Rapid dynamic changes of FL.2 variant: A case report of COVID-19 breakthrough infection
Source: Int J Infect Dis. 2024 Jan;138:91–6. doi: 10.1016/j.ijid.2023.11.011 (PMC10719116; doi:10.1016/j.ijid.2023.11.011)
Supplement: Supplementary file 1 [file mmc1.docx]

**
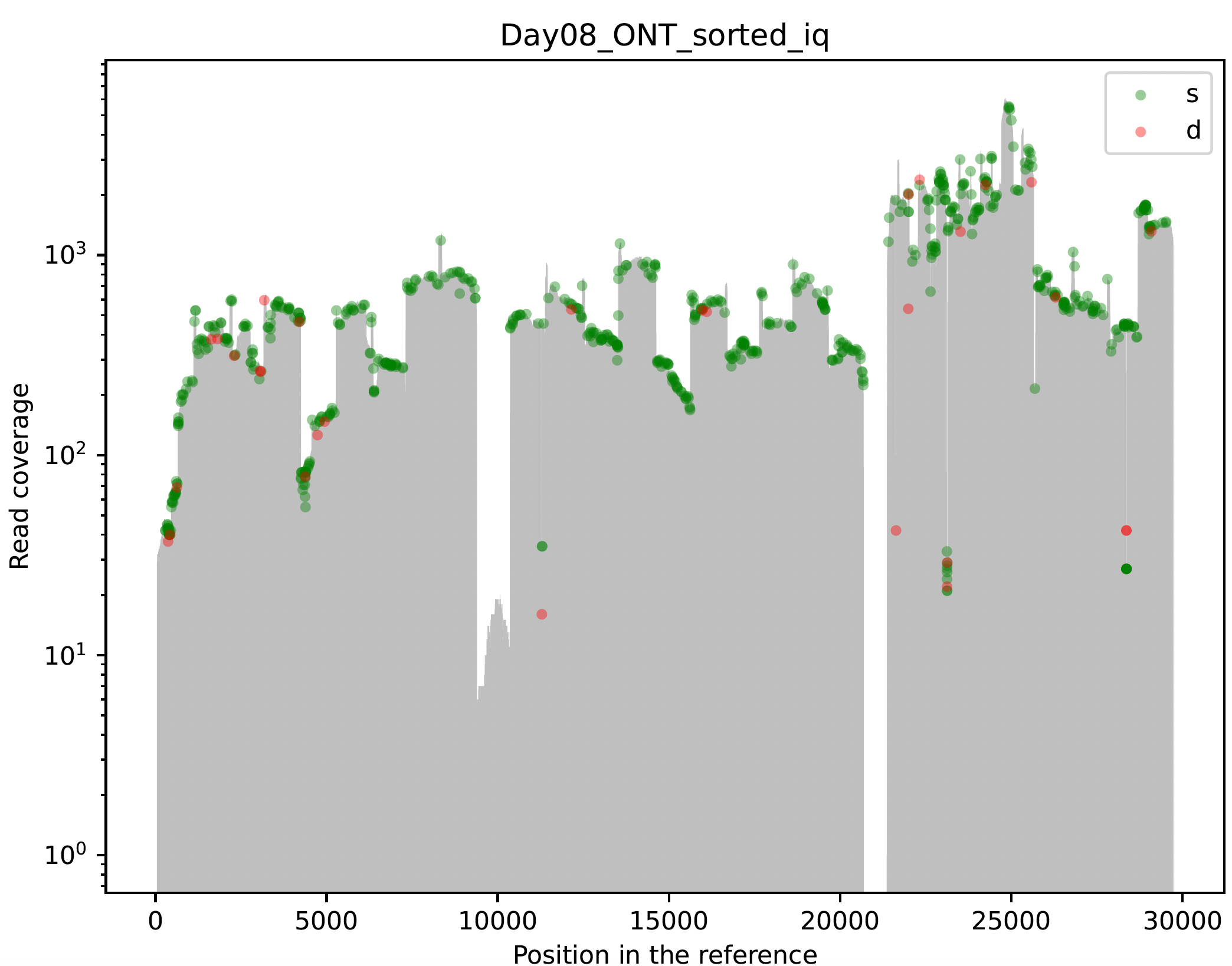

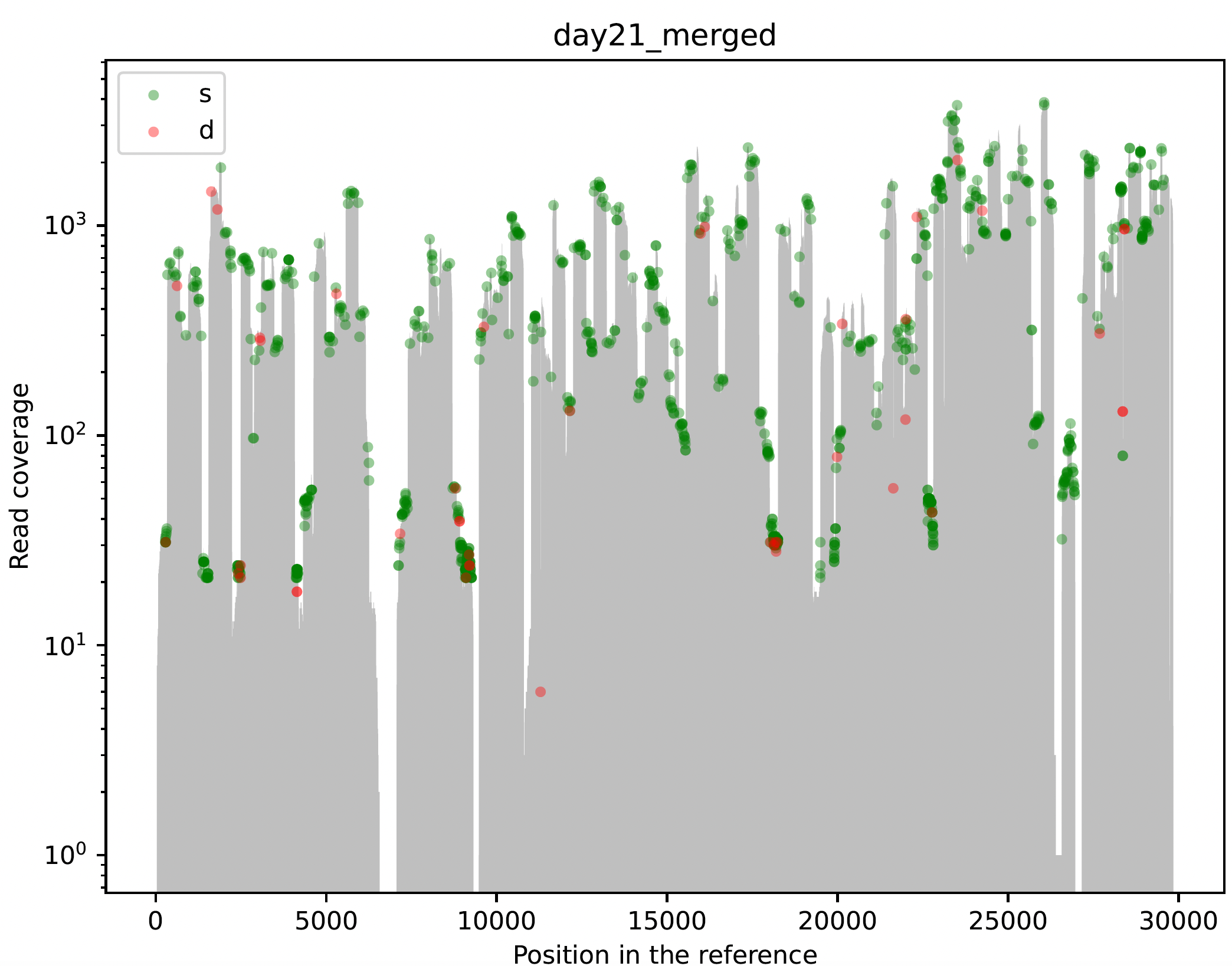
**

**Supplementary Figure S1:** Genome coverage profiles for the three nasopharyngeal swabs analyzed in this study. Y-axis is read coverage scale. A) Day-08 sequence; B) Day-21 sequence. *s* represents substitions; *d* represents deletions.
