# Supplementary material for: Rapid dynamic changes of FL.2 variant: A case report of COVID-19 breakthrough infection
Source: Int J Infect Dis. 2024 Jan;138:91–6. doi: 10.1016/j.ijid.2023.11.011 (PMC10719116; doi:10.1016/j.ijid.2023.11.011)
Supplement: Supplementary file 2 [file mmc2.docx]

**Supplementary Figure S2:** Spike protein mutation profiles of Day-08 (A) and Day-21 (B) consensus sequences. Mutations in Day-08 sequences are **nsp1:** K47R, G112S, S135R, **Plpro:** T24I, G489S, T577I, G1001S, **nsp4:** T327I, L438F, T492I, **3CLpro:** P132H, **nsp9:** T35I, **RdRP:** P323L, G671S, **nsp13:** S36P, R392C, **nsp14:** I42V, **nsp15:** T112I, **Spike:** T19I, V83A, G142D, H146Q, Q183E, V213E, G252V, G339H, R346T, L368I, S371F, S375F, T376A, D405N, R408S, **K417N**, N440K, V445P, G446S, N460K, S477N, T478K, E484A, F486P, F490S, Q498R, N501Y, Y505H, D614G, H655Y, N679K, P681H, N764K, D796Y, Q954H, N969K, **ORF3a:** T223IE: T9I, T11AM, Q19E, A63T, S136N, **ORF6:** D61L, **ORF8:** G8*, **N:** P13L, R203K, G204R, and S413R. Mutations in Day-21 sequences are **nsp1:** K47R, S135R, **PLpro**:T24I, G489S, G1001S, **nsp4:** T327I, L438F, T492I, **3CLpro**: P132H, **nsp9**:T35I, **RdRP**: P323L, G671S, **nsp13**S36P, R392C, **nsp14:** I42V, **nsp15**: T112I, **Spike**: T19I, V83A, G142D, H146Q, Q183E, V213E, G252V, **P330S**, G339H, R346T, L368I, S371F, S373P, S375F, T376A, D405N, R408S, N440K, V445P, G446S, N460K, S477N, T478K, E484A, F486P, F490S, Q498R, N501Y, Y505H, D614G, H655Y, N679K, P681H, N764K, D796Y, Q954H, N969K, **ORF3a**: T223I, ET9I, T11AM, K14K*EQ, Q19IKRT, A63T, S136N, G157G*R, K180KN, L181LMV, A188T, S197SCGR, **ORF6**: D61L, ORF8, G8*, **N**: P13L, R203K, G204R, and S413R.
