# Supplementary material for: Rapid dynamic changes of FL.2 variant: A case report of COVID-19 breakthrough infection
Source: Int J Infect Dis. 2024 Jan;138:91–6. doi: 10.1016/j.ijid.2023.11.011 (PMC10719116; doi:10.1016/j.ijid.2023.11.011)
Supplement: Supplementary file 3 [file mmc3.docx]

**Supplementary Table 1**: Distribution of minority variants in the S protein between 2 time points.

| **Mutation** | | **G142D** | | **P330S** | | **K417N** | |
| --- | --- | --- | --- | --- | --- | --- | --- |
| Nucleotide Change | | 21987:G>A | | 22550:C>T | | 22813:G>T | |
| Codon | REF | GGT | | CCT | | AAG | |
|  | ALT | GAT | | TCT | | AAT | |
| **Sequence** | | **Day-08** | **Day-21** | **Day-08** | **Day-21** | **Day-08** | **Day-21** |
| Count | REF | 699 | 205 | 1590 | 973 | 1427 | - |
|  | ALT | 1282 | 126 | 215 | 700 | 367 | ~~-~~ |
| Proportion | REF | 34.33% | 59.08% | 86.85% | 44.92% | 78.49% | - |
|  | ALT | 62.97% | 36.31% | 8.32% | 52.63% | 20.19% | - |
| Statistical comparison | | p<0.05 | | p<0.05 | | p<0.01 | |
